# Molecular Cloning of a New Immunomodulatory Protein from *Anoectochilus formosanus* which Induces B Cell IgM Secretion through a T-Independent Mechanism

**DOI:** 10.1371/journal.pone.0021004

**Published:** 2011-06-16

**Authors:** Yen-Chou Kuan, Tsai-Jen Wu, Che-Yu Kuo, Ju-Chun Hsu, Wen-Ying Chang, Fuu Sheu

**Affiliations:** 1 Department of Horticulture, National Taiwan University, Taipei, Taiwan, Republic of China; 2 Center for Biotechnology, National Taiwan University, Taipei, Taiwan, Republic of China; Agency for Science, Technology and Research - Singapore Immunology Network, Singapore

## Abstract

An immunomodulatory protein (IPAF) was purified and cloned from *Anoectochilus formosanus*, an Orchidaceae herbal plant in Asia. The major targeting immune cells of IPAF and its modulating effects toward B lymphocytes were investigated. Rapid amplification of cDNA ends (RACE) was conducted to clone the IPAF gene, and the obtained sequence was BLAST compared on the NCBI database. MACS-purified mouse T and B lymphocytes were stimulated with IPAF and the cell proliferation, activation, and Igs production were examined. IPAF comprised a 25 amino acids signal peptide and a 138 amino acids protein which was homologous to the lectins from Orchidaceae plant. IPAF selectively induced the cell proliferation in mouse splenic B lymphocytes but not T lymphocytes. The IPAF-induced B cells exhibited increased CD69 and MHC class II expression, and a dose- and time-dependent enhancement in IgM production. These results suggested potential benefits of IPAF to strengthen the humoral immunity.

## Introduction

*Anoectochilus formosanus* is an herbal plant widely used as dietary supplement and folk remedy in Asia. It provided numerous health benefits such as hepatoprotection [1]–[4], anti-fatigue [5], anti-oxidative [6], [7], anti-hyperglycemia [8], anti-hyperliposis [4], anti-osteoporosis [9], [10], anti-tumor [11], [12], and immune modulation. The aqueous extraction of *A. formosanus* was reported to stimulate lymphoid tissue and peritoneal macrophages [11]. Another study using an ovalbumin-inhaled murine model indicated the administration of *A. formosanus* extract was capable of modulating cytokine secretion and regulatory T cell population in bronchoalveolar lavage fluid [13]. However, the bioactive components of *A. formosanus* are still unclear.

B lymphocytes are the central mediator of humoral immunity and play a pivotal role in host defense. Upon encountering with antigen through B cell receptors (BCR), B cells could proliferate and differentiate into effector plasma cells through a highly regulated process and neutralized pathogens by secreting antigen-specific antibodies [14]. B cells are antigen presenting cells capable of breaking down antigens into small peptide fragments which are presented on the major histocompatibility complex (MHC) located on the surface of B cells [15], [16], thus allowing recruitment of cognate CD4^+^ T helper cells to facilitate the complete activation of B cells. Antigens recognized by B cells can be divided into two categories, T cell-dependent (TD) antigen and T cell-independent (TI) antigen. The latter can be further categorized into TI type-1 (TI-1) and TI type-2 (TI-2) antigens [17]. B cells activated by TD antigens such as tetanus, diphtheria, and pertussis toxin require cognate help from T cells for complete activation. TI-1 antigen such as LPS or bacterial DNA can activate B cell through antigen-BCR interactions and additional signals provided by other receptors such as Toll-like receptors (TLRs). TI-2 antigen such as multivalent polysaccharide or antigen with repetitive structure could activate antigen-specific B cells through extensive cross-linking of BCRs [18].

In the current study, we purified a novel protein from fresh A. formosanus. To characterize this protein, its N-terminal amino acid sequence was determined and its cDNA sequence was cloned using RACE method. Moreover, we observed that this protein was capable to induce mouse peritoneal macrophages and splenic lymphocytes and designated it as an immunomodulatory protein from A. formosanus (IPAF). The IPAF-stimulated cell activation and maturation of mouse B lymphocytes were investigated. The necessity of T lymphocytes and the involvement of TLR2 and TLR4 within this induction were also evaluated. To our knowledge, IPAF is the first Orchidaceae protein reported to activate B lymphocytes as a TI antigen.

## Results

### Purification and biochemical characteristics of IPAF

**Figure 1:**
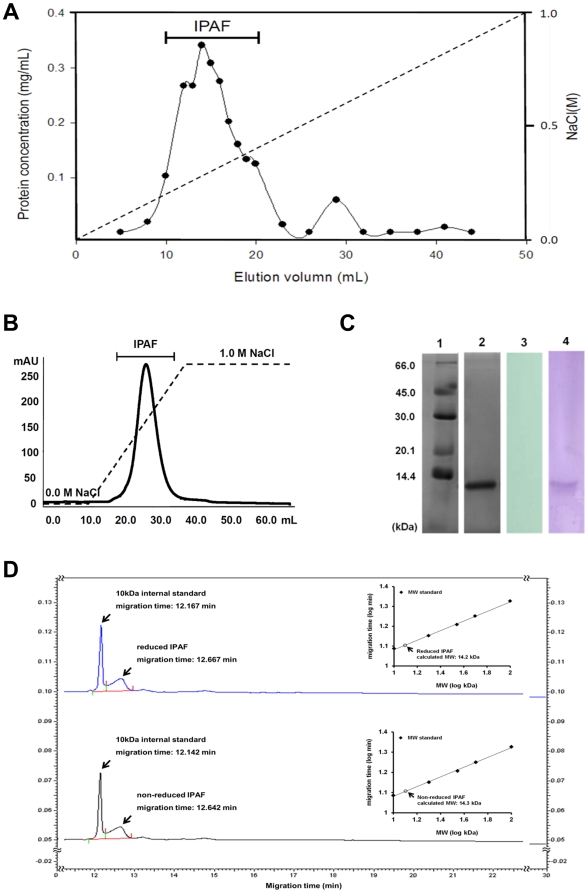
Purification and biochemical characteristics of IPAF. (A) Crude protein extracts were fractionated by a DEAE-52 cellulose column eluted with linear gradient of 0.0–1.0 M NaCl. The elution profile was generated based on protein concentration determined by BCA analysis. (B) The active fractions were further purified using FPLC system equipped with a HiTrapQ anion exchange column eluted with linear gradient of 0.0–1.0 M NaCl. The elution profile was generated by measuring the absorbance at 280 nm. (C) Purified IPAF was identified by SDS-PAGE with Coomassie brilliant blue (lane 2) and periodic acid-Schiff staining (lane 3). Purified IPAF was transferred to PVDF membrane and identified via western blot using a self-made mAb against IPAF (lane 4). The molecular weight of IPAF was determined by comparing with pre-stained protein markers (lane 1). (D) Gel-filtration capillary electrophoresis SDS-MW analysis of IPAF prepared under reducing or non-reducing condition. The MW was derived by normalizing the migration time of the samples with the 10 kDa internal standard, and calibrated with the standard curve constructed with the protein size standard.

To isolate the novel protein from *A. formosanus*, the crude protein solution was obtained by means of homogenization, sonication and, precipitation. The total protein yield was 1.67 g crude protein per kg plant weight. Subsequently, the crude protein extract was fractionated using a DEAE-52 cellulose column eluted with linear gradient of 0.0–1.0 M NaCl. The elution profile acquired by BCA protein concentration analysis exhibited one major peak (Fig. 1A), which was collected to determine the stimulatory activity toward mouse RAW264.7 macrophage cells. Remarkable activity was observed in view of significant increase in TNF-α production in RAW264.7 (Supporting Information S1). The SDS-PAGE analysis of these fractions revealed two protein bands (Supporting Information S2). The fractions were pooled for protein yield calculation (0.25 g per kg plant weight) and further purification with the fast protein liquid chromatography (FPLC) system. The sample was loaded on HiTrapQ anion exchange column and eluted with a linear gradient of 0.0–1.0 M NaCl. The elution profile was obtained by measuring the absorbance at 280 nm and revealed a single peak (Fig. 1B). The fractions corresponding to this peak were pooled for protein yield calculation (86.7 mg per kg plant weight) and SDS-PAGE analysis. A single 14 kDa protein band could be observed after CBR staining (Fig. 1C). The SDS-PAGE was also stained with PAS reagent for carbohydrate content determination, where the result suggested that this protein is not a glycoprotein (Fig. 1C). This 14 kDa protein was capable of up-regulating the TNF-α and IFN-γ secretion by RAW264.7 cells and murine splenocytes, respectively (Supporting Information S3, S4), and was then believed to be an immunomodulatory protein from Anoectochilus formosanus (IPAF). Viewing on the fact that the IPAF sample examined in SDS-PAGE was reduced, it was possible that the observed 14 kDa band might be a subunit if the protein constituted two or more subunits. To investigate whether IPAF was a monomeric protein, we compared the MW of reduced and non-reduced IPAF by gel-filtration capillary electrophoresis. The non-reduced IPAF sample was prepared under conditions where the intermolecular bondings such as disulfide bonds and hydrogen bonds were preserved. As shown in Fig. 1D, the migration time of reduced and non-reduced IPAF were very close, and the MW, as calculated with protein size standard curve, were 14.2 and 14.3 kDa for reduced and non-reduced IPAF, respectively. Since the intermolecular bondings were saved in the non-reduced sample, the MW would have been larger if it constituted more subunits. This finding confirmed that IPAF was a monomeric protein, and the 14 kDa protein band observed on SDS-PAGE was indeed IPAF. To facilitate future research, we used immunized mouse splenocytes and fused them with mouse leukemia cell line X63 to generate monoclonal hybridoma secreting antibodies specifically against IPAF. The specificity of the monoclonal antibody was validated via Western blot analysis (Fig. 1C).

### Molecular cloning of IPAF

**Figure 2:**
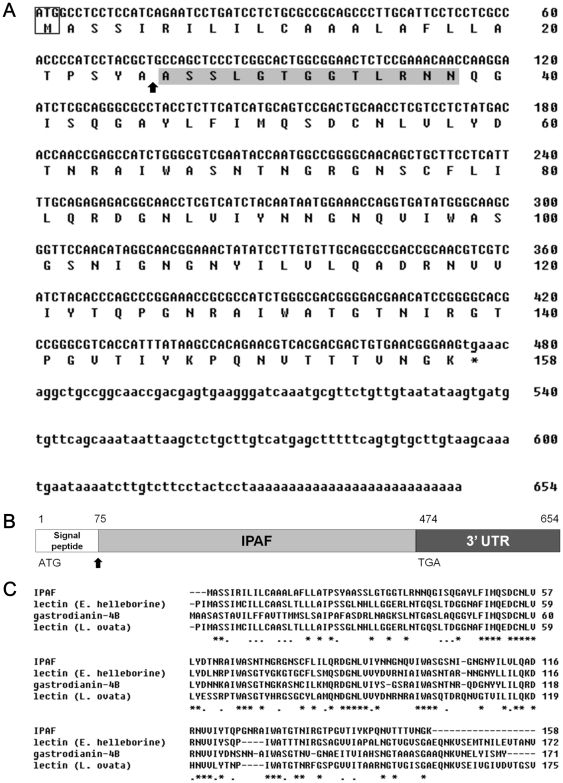
Molecular cloning of IPAF. (A) Complete nucleotide sequence of IPAF cDNA was derived by RACE PCR using specific primers of IPAF. The ORF of IPAF cDNA is presented in capital letters, the termination codon is marked with an asterisk, and the 3′-UTR is displayed in lower case letters. In the amino acid sequence, the putative signal peptide cleavage site between Ala25 and Ala26, as predicted by SignalP, is showed with a vertical arrow, and the N-terminal amino acid sequence is indicated with a grey background. (B) Genomic structure of IPAF gene. The numbers represent base pair positions in the genomic sequence. The putative signal peptide, IPAF gene, and 3′-UTR are presented with white, grey, and dark-grey background, respectively. The vertical arrow indicates the corresponding cleavage site of the signal peptide. (C) Multiple alignment of IPAF amino acid sequence with sequences of lectins from Orchidaceae plants was performed by ClustalW2 program on the website of EMBL-EBI. The identical and the similar residues are marked with asterisks and dots, respectively.

To obtain the amino acid sequence of IPAF, purified IPAF separated by SDS-PAGE was transferred onto a PVDF membrane and subjected to Edman degradation for N-terminal sequence determination. The resulted sequence, ASSLGTGGTLRNN, did not belong to any known protein under the BLAST search of NCBI database. Additionally, the lack of methionine at the N-terminus suggested that the mRNA-translated IPAF could contain a hydrolysable signaling sequence in its amino terminal side. Taking advantage of the N-terminal amino acid sequence of IPAF, we adopted the RACE approach to clone the IPAF gene. The 3′-RACE was performed using a gene-specific primer a36, and a 700 bp DNA fragment (designated as PRa36) was attained. The N-terminal amino acid sequence of an ORF of this DNA fragment was fully identical to the sequence of IPAF (Supporting information S5), suggesting that PRa36 was a candidate for the 3′ portion of the IPAF gene. To achieve the complete cDNA of IPAF, a primer (b18) with a sequence from nucleotide position 358 to 378 of the PRa36 fragment was further designed and utilized to conduct the 5′-RACE PCR, by which a 600 bp DNA fragment (PRb18) was generated. The 26^th^ to 38^th^ amino acid residues of translated PRb18 sequence were identical to the N-terminal amino acid sequence of IPAF (Supporting Information S5). By merging the corresponding two ORFs of PRa36 and PRb18, the complete cDNA sequence of IPAF was obtained (Fig. 2A). It was 654 bp in length, including 474-bp ORF encoding 158 amino acids and 151-bp 3′-UTR. The first 25 amino acid residues were putative signal peptide (possibility of ∼0.90) as predicted by the SignalP program (Supporting information S6). The peptide may be cleaved during post-translational modification or secretion of IPAF (Fig. 2B). The nucleotide sequence of IPAF has been deposited in the GenBank® database under accession no. ADK74829.

### Homology analysis of IPAF to Orchidaceae lectins

Comparisons between the amino acid sequence of IPAF and other sequences using the protein BLAST program via NCBI revealed a close relation between IPAF and the plant lectin of *Epipactis helleborine* (AAA19577.1), gastrodianin-4B of *Gastrodia elata* (AAX10109.1), and lectin of *Listera ovata* (AAA20899.1). Multiple alignment of IPAF amino acid sequence with these proteins (Fig. 2C) displayed considerable sequence identities and similarities as compiled in Table 1. Notably, *E. helleborine*, *G. elata* and *L. ovata* all belong to the Orchidaceae family. The hemagglutinating activity of IPAF was also examined and revealed that IPAF, at 1.0 mg/mL or below, agglutinated neither mouse nor human erythrocytes (Supporting information S7). This result coincide with the discovery of Van Damme et al. [19] where one class of lectins devoid of erythrocyte agglutinating activity was isolated from *L. ovata* and *E. helleborine*. Hence, these findings suggested the close relation between IPAF and Orchidaceae lectins.

**Table 1:** Amino acid sequence comparison of IPAF with lectins from Orchidaceae plants.

Species	Name	Access No.	Length	Identity(%)	Similarity(%)	Gap(%)
*A. formosanus*	IPAF	ADK74829.1	158	-	-	-
*E. helleborine*	lectin	AAA19577.1	172	57	68	2
*G. elata*	gastrodianin-4B	AAX10109.1	171	51	66	1
*L. ovata*	lectin	AAA20899.1	175	51	65	3

a) The identity values were calculated as (numbers of amino acid residues identical to IPAF/numbers of IPAF amino acid residues).

b) The similarity values were calculated as (numbers of amino acid residues similar to IPAF/numbers of IPAF amino acid residues).

c) The gap values were calculated as (numbers of gaps/numbers of IPAF amino acid residues).

### B lymphocytes as the major targets activated by IPAF

**Figure 3:**
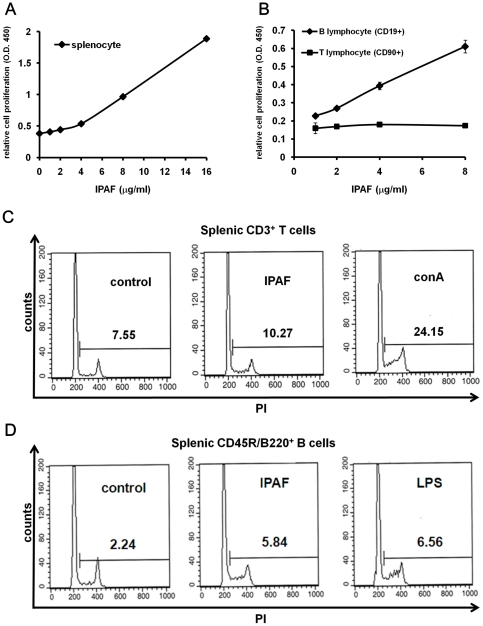
IPAF stimulated the cell proliferation of mouse splenic B lymphocytes. (A) Splenocytes harvested from BALB/c (5×10^5^ cells per well) were treated with IPAF of indicated concentrations for 72 hours, cell proliferation was determined by BrdU assay. (B) MACS purified splenic T (CD90^+^) cells and B (CD19^+^) cells from BALB/c were treated with IPAF of indicated concentrations for 72 hours, cell proliferation was determined by BrdU assay. (C) Splenocytes harvested from BALB/c were treated with medium (left), 8 µg/mL IPAF (middle), 5 µg/mL conA (right) for 72 hours. Cells were harvested and stained with PE-labeled anti-mouse CD3 and PI for flow cytometry analysis. T cells were gated based on CD3 fluorescence, and proliferating cells were determined based on PI fluorescence intensity. Data are presented as percentage of proliferating cells in total cells. (D) Splenocytes harvested from BALB/c were treated with medium (left), 8 µg/ml IPAF (middle) and 5 µg/mL LPS (right) for 72 hours. Cells were harvested and stained with FITC-labeled anti-mouse CD45R/B220 and PI for flow cytometry analysis. B cells were gated based on CD45R/B220 fluorescence, and proliferating cells were determined based on PI fluorescence intensity. Data are presented as percentage of proliferating cells in total cells.

Preliminary studies of IPAF have revealed the capability of IPAF in activating mouse splenocytes (Supporting Information S4). We therefore examined the major target cells regulated by IPAF. After 72 hours of induction, a remarkable and dose-dependent increase in cell proliferation by mouse splenocytes was observed, indicating that IPAF (1–16 µg/mL) was capable of enhancing mouse splenocytes proliferation (Fig. 3A). It has been known that splenocytes were mainly consisted of T and B lymphocytes, which played crucial roles in immune responses. We further determine the stimulatory effect of IPAF on mouse T and B lymphocytes. The primary T and B lymphocytes (CD90^+^ and CD19^+^ cells), which were positively selected from splenocytes of BALB/c with cell purity above 95%, were co-cultured with IPAF (1–16 µg/mL) for 72 hours. The cell proliferation was then determined by means of BrdU assay, and the results demonstrated that IPAF stimulated CD19^+^ B lymphocytes in cell proliferation directly and dose-dependently (Fig. 3B). Nevertheless, this effect was absent in IPAF treated T lymphocytes, suggesting T lymphocytes should not be the main responding cells induced by IPAF (Fig. 3B). In accordance with this observation, the result of FACS assay demonstrated that IPAF exhibited minor stimulating effect on T lymphocyte proliferation (Fig. 3C). The cell proliferation level of IPAF-induced B lymphocytes, however, markedly increased by 2.61-fold as compared with control (Fig. 3D). Taken together, these data suggested that B lymphocytes could be the main targets to be activated by IPAF.

### IPAF stimulated activation and maturation of B lymphocytes

**Figure 4:**
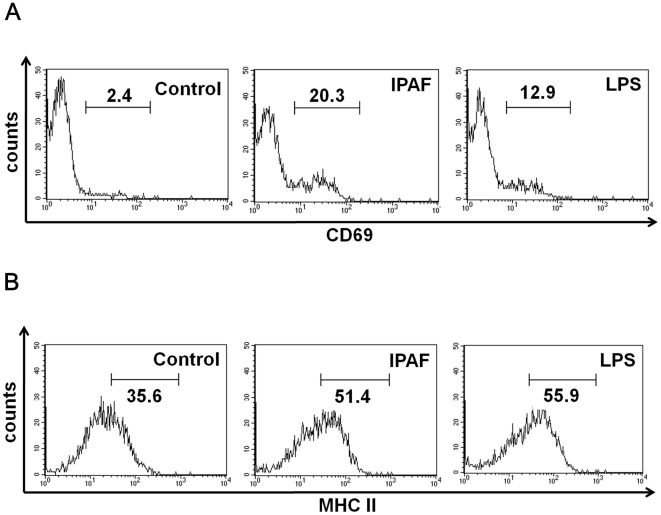
IPAF stimulated the expression markers in mouse splenic B lymphocytes. (A) Splenic B lymphocytes of BALB/c were stimulated with 30 µg/mL IPAF (middle), 10 µg/mL LPS (right), or medium alone (left) for 24 hours. Cells were stained with PE-labeled anti-mouse CD69 for flow cytometry analysis. Data are presented as percentage of CD69 positive cells in total cells. (B) Splenic B lymphocytes of BALB/c were stimulated with 30 µg/mL IPAF (middle), 10 µg/mL LPS (right), or medium alone (left) for 24 hours. Cells were stained with FITC-labeled anti-mouse MHC class II for flow cytometry analysis. Data are presented as percentage of MHC class II positive cells in total cells.

Based on the findings that B lymphocytes were the main cells stimulated and proliferated by IPAF, whether IPAF caused the cell activation and development of mouse splenic B lymphocytes or not was evaluated using the FACS approach. The lymphocyte activation marker CD69 expression by MACS purified IPAF-induced B lymphocytes was examined, and an 8.46-fold elevation in CD69 expression was attained (Fig. 4A). In addition, B lymphocytes are antigen presenting cells that bridged the innate and adaptive immunity, therefore, antigen presenting ability of IPAF-treated B lymphocytes was investigated. The MHC class II expression was 1.44-fold higher in IPAF-induced B lymphocytes than in untreated cells (Fig. 4B). These data indicated that IPAF was capable of activating the development and maturation of mouse splenic B lymphocytes and strengthen their antigen presenting ability by elevating MHC class II expression.

### IPAF enhanced IgM but not IgG production in B lymphocytes

**Figure 5:**
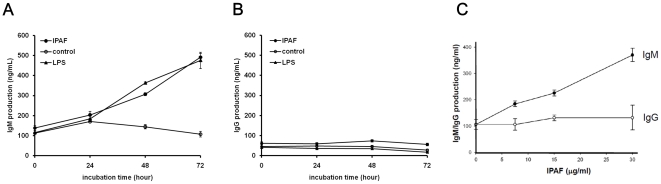
IPAF enhanced IgM production in purified mouse B lymphocytes. (A) IgM production and (B) IgG production by MACS-purified CD19^+^ B lymphocytes of BALB/c were treated with 30 µg/mL of IPAF (•), medium (○), or 10 µg/ml LPS (▴) for 0, 24, 48 and 72 hours. The Igs production was determined by ELISA. Results are shown as mean ± SD in triplicate (n = 3). (C) B lymphocytes of BALB/c were treated with IPAF of indicated concentrations for 48 hours. IgM (•) and IgG (○) production were determined by ELISA. Results are shown as mean ± SD in triplicate (n = 3).

The sole weapon of B lymphocytes against microorganism pathogens is the secretion of neutralizing antibodies. The capability of IPAF alone in modulating antibody production in mouse splenic B lymphocytes was determined by ELISA quantification methods. MACS purified IPAF-treated B lymphocytes exhibited consistent and time-dependent enhancement in IgM production (Fig. 5A). As fully activated B lymphocytes would undergo Igs isotype switch and shift from IgM secreting plasma cells to IgG secreting plasma cells, the levels of IgG in the cell culture supernatant were measured. It was observed that IgG production was not affected by IPAF within 72 hours of induction (Fig. 5B). We then investigated the dose effect of IPAF on the antibody production by the B lymphocytes and revealed that IgM production by IPAF-treated B lymphocytes increased dose-dependently, while IgG production was unaffected (Fig. 5C). These data confirmed that IPAF was capable to enhance IgM production by B lymphocytes in time- and dose-dependent manners.

### IPAF-induced B lymphocyte activation was TLR2/TLR4 independent

**Figure 6:**
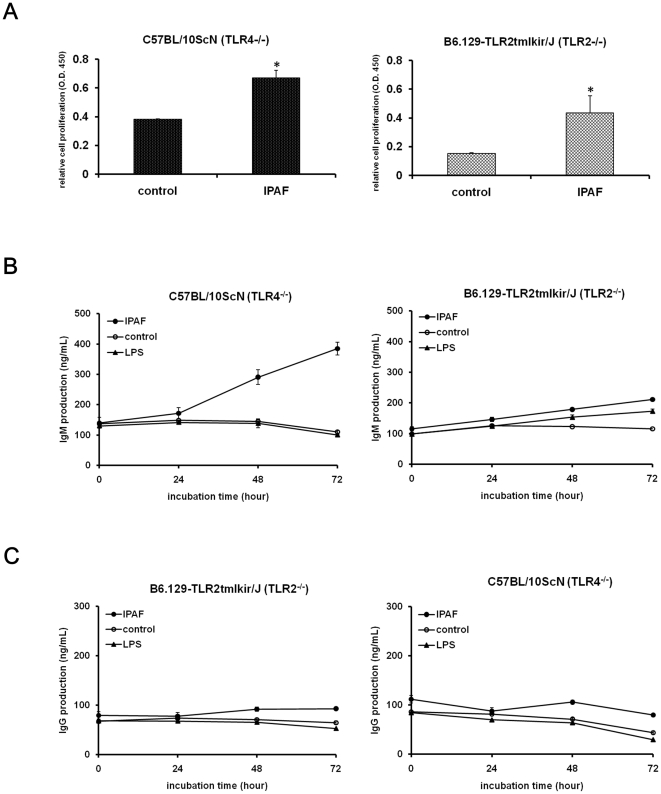
TLR2 and TLR4 were not involved in the IPAF-induced activation of mouse splenic B lymphocytes. (A–B) B lymphocytes of C57BL/10ScN (TLR4^−/−^) and B6.129-TLR2tmlkir/J (TLR2^−/−^) were treated with 30 µg/mL of IPAF (•), medium (○), or 10 µg/mL LPS (▴) for 0, 24, 48 and 72 hours, the IgM (A) and IgG (B) production were determined by ELISA. (C) B lymphocytes of C57BL/10ScN and B6.129-TLR2tmlkir/J were treated with or without 30 µg/ml IPAF for 48 hours, and the cell proliferation was analyzed by Brd-U assay. Results are shown as mean ± SD in triplicate (n = 3). *P<0.05 versus control.

Several studies have described the possible involvements of TLRs in the activation of B lymphocytes [20]–[22]. In preliminary studies, we have discovered that IPAF stimulated mouse primary peritoneal macrophages mainly through TLR4 (Supporting information S8). To investigate the involvement of TLR2/TLR4 in IPAF-induced B lymphocyte activation, we purified splenic B lymphocytes from C57BL/10ScN (TLR4^−/−^) and B6.129-TLR2tmlkir/J (TLR2^−/−^), and evaluated their responses to IPAF. As shown in Fig. 6A, the stimulating effect of IPAF on cell proliferation was significant both in C57BL/10ScN and B6.129-TLR2tmlkir/J B lymphocytes. We further examined the elevating effect of IPAF on CD69 and MHC class II expressions by C57BL/10ScN and B6.129-TLR2tmlkir/J B lymphocytes and found that the expression levels of CD69 and MHC class II were significantly upregulated despite the lack of TLR4 and TLR2 receptors (Supporting Information S9, S10, S11, S12). After the analysis of the IgM and IgG production level of IPAF treated C57BL/10ScN and B6.129-TLR2tmlkir/J B lymphocytes, similar results where IgM production was significantly increased in a time dependent manner was observed (Fig. 6B), and IgG production was unaffected (Fig. 6C). Based on these findings, we suggested that neither TLR4 nor TLR2 were the major receptor of IPAF to exert the stimulating effects in mouse splenic B lymphocytes.

## Discussion

In this study, IPAF which possessed significant stimulating effect toward mouse splenic B lymphocyte was purified and cloned. To our knowledge, it is the first report to characterize an immunomodulatory protein of the Orchidaceae plant *A. formosanus*. The MW of IPAF determined by SDS-PAGE (∼14 kDa) and gel-filtration capillary electrophoresis (14.3 kDa) agreed well with the MW deduced from the amino acid sequence (14.4 kDa). The discovery of non-reduced and reduced IPAF possessing nearly identical MW also confirmed that IPAF was a monomeric protein. IPAF was a non-glycosylated protein and the translated IPAF contained putative signal peptide of 25 residues, which might be cleaved in post-translational modification or secretion. The mature IPAF encoded 133 amino acids with a MW of 14 kDa, which was similar to those of lectins (∼13 kDa) isolated from *L. Ovata*, *E. helleborine* and *Cymbidium* hybrid [23]. The MW of the IPAF putative signal peptide was 2.45 kDa based on its amino acid sequence. Notably, the cDNA sequences of lectins of *L. Ovata*, *E. helleborine* and *Cymbidium* hybrid all contained a 2.5 kDa signal peptide [24]. Protein BLAST comparison and multiple alignment of the amino acid sequence of IPAF within NCBI database revealed its high similarity to the lectins from Orchidaceae plants, including *E. helleborine* lectin (AAA19577.1), gastrodianin-4B of *G. elata* (AAX10109.1), and lectin of *L. ovata* (AAA20899.1). Although the physiological functions of *E. helleborine* and *L. ovata* lectins were not well understood, Gastrodianin-4B which abundantly expressed in the above-ground organs of *G. elata* was considered as an important defensive protein against fungus *Armillaria*. Its elevated expression in the aerial part of the plant was possible to be responsible for the defenses against other plant pathogens [25]. Based on the high sequence similarity between IPAF and gastrodianin-4B, and the fact that the above-ground organs of *A. formosanus* contributed more than 80% of the processed plant weight, the physiological function of IPAF in *A. formosanus* was assumed to resemble gastrodianin-4B, as a defensive molecule against phytopathogens. Our hemagglutination assay showed that IPAF possessed no agglutinating activity toward human and mouse erythrocytes. Interestingly, Van Damme et al. [19] also discovered a group of lectins from *E. helleborine* and *L. ovata* which were unable to agglutinate erythrocytes. In that study, it was suggested that the monomeric nature of those lectins might be the reason of their inability to agglutinate erythrocyte. This again was parallel to our finding where IPAF was a monomeric protein and agglutinated neither human nor mouse erythrocytes. According to the study of Van Damme et al., lectins are ubiquitous in different tribes of the family of Orchidaceae [23]. Based on our discoveries, we believed that IPAF was a new lectin or a lectin-like protein of the Orchidaceae lectin family.

Lectins are ubiquitous in nature, and in the higher plant, myriad of them are known to possess immunostimulating activity. For examples, lectins from mistletoe (*Viscum album*) were reported to be capable of elevating the population and activity of murine natural killer cell both *in vitro* and *in vivo* [24]; lectins from garlic (*Allium sativum*) were reported to proliferate murine thymocytes and splenocytes [26]; banana lectin from *Musa pradisiac* exhibited proliferation potential in CD3^+^, CD4^+^, and CD8^+^ cell populations of human PBMCs [27]; lectin from *Narcissus tazetta* displayed capability of activating murine macrophage and splenocytes [28]; lectin from the seeds of *Momordica charantia* activated murine B lymphocytes and induced their polyclonal IgM secretion in a T cell independent manner [29]. Since there hasn't been any report of Orchidaceae lectin regarding the immunomodulatory efficacy, this is the first article to elucidate the immunological function of an Orchidaceae lectin-like protein, IPAF, as an activator of murine B lymphocyte.

We discovered that B lymphocyte was the major target cell responsible to IPAF induced cell proliferation. IPAF could directly stimulate the purified B (CD19^+^) cells in the absence of T cells. Moreover, IPAF was capable of activating the development and maturation of purified B lymphocytes in view of the increased expression of CD69 and MHC class II, and the significant enhancement of IgM production. This characteristic selective activation of B lymphocytes by IPAF resembled that of the lectin isolated from *Momordica charantia*, which also specifically activated murine B lymphocytes, possibly through selective binding of cell specific carbohydrate [29]. Another similar mitogenic substance, which primarily activated B cells, inducing the cell proliferation and IgM secretion was found in the seeds of *Ulex europeus* [30]. Still another pure B cell activator was *Staphylococcus aureus* Cowan I (SAC), which acted on B cells by cross-linking the Igs receptors on the B cell surface and triggered the cell proliferation [31]. It was exhibited that IPAF followed an analogous activation pattern of *Momordica charantia* lectin, *Ulex* mitogen, or SAC, in which B lymphocytes were immediately stimulated without the aid from T lymphocytes or any other accessory cells. Furthermore, it was believed that typical TI-2 antigens acted by cross-linking BCRs and co-receptors through their highly repetitive epitopes such as cell-wall polysaccharides of pneumococcus (*Streptococcus pneumoniae*). Considering the fact that IPAF was a purified soluble protein possessing none of the characteristics of any typical TI-2 antigens, we assumed that IPAF might be a TI-1 antigen, which triggered B cell differentiation to IgM-secreting plasma cells through BCR ligation along with additional stimulations provided by other signaling molecules [32]. Additional researches are required to clarify the above assumption. On the other hand, the cytokines and molecular signals provided by the effector T helper cells were required for the isotype switching of antibodies secreted by B lymphocyte [32]. It was reasonable to discover that the IgG production of purified B cells was scarcely affected by IPAF induction.

Preliminary experiments indicated that TLR4 could be the dominating receptor of IPAF on murine peritoneal macrophages (Supporting Information S8). TLRs are pattern recognition receptors in sensing molecules and could deliver stimulating signals toward B lymphocytes [20]–[22]. Therefore, TLRs (especially TLR4 and TLR2 recognizing the peptidoglycan and protein ligands, respectively) were possible responding molecules for IPAF in murine B lymphocytes. However, the significant stimulative effect of IPAF on the cell proliferation and IgM production still persisted in the B cells of both TLR4^−/−^ (C57BL/10ScN) and TLR2^−/−^ (B6.129-TLR2tmlkir/J) mice. Based on these results, it was inferred that neither TLR4 nor TLR2 were the major receptor of IPAF, notwithstanding, further investigation was required to elucidate the receptor and the signaling network of B cell activation induced by IPAF.

In conclusion, this study is the first to purify and clone an immune regulator protein from *A. formosanus*. IPAF was capable of activating the cell proliferation, maturation, and IgM production by mouse splenic B lymphocytes in a T cell independent manner through a TLR2/4 independent mechanism. Our observations provided new insight to discover functional protein from Orchidaceae plant regarding the immunomodulating ability toward mammalian cells.

## Materials and Methods

### Purification of IPAF

Tissue cultured *Anoectochilus formosanus* plant was purchased from Yo-Rong farm (Puli, Taiwan). The whole plants were cleaned, minced, weighed and then immersed in equal weight of extraction buffer containing 5% (v/v) acetic acid, 0.1% (v/v) 2-mercaptoethanol, and 0.308 M sodium chloride. The samples were then homogenized using a Waring Laboratory blender (Waring Laboratory, Torrington, WY) and subjected to sonication by Sonicator XL2015 (MISONIX, Farmingdale, NY) to yield plant extracts. The extracts were subsequently sieved with a screen mesh and centrifuged at 10,000 rpm at 4°C for 1 hour to remove plant residuals and debris. Supernatant containing crude protein was treated with 30% saturation of ammonium sulfate and left standing for 24 hours for protein precipitation. After precipitation by ammonium sulfate, crude protein solution was centrifuged at 10,000 rpm at 4°C for 1 hour, and the precipitates were collected, suspended and dialyzed in 0.01 M Tris-HCl buffer (pH 8.2) for 72 hours with at least six changes of dialysis solution. The dialysate was then loaded on a DEAE 52 cellulose (Whatman, Maidstone, UK) column (2.5×20 cm) pre-equilibrated with 0.01 M Tris-HCl buffer (pH 8.2) for fractionation. The column was eluted with linear gradient of 0.0–1.0 M NaCl. The main active fractions (Fig. 1A) were pooled and further purified by FPLC system with a HitrapQ anion exchange column (GE Healthcare, Buckinghamshire, UK) eluted with a linear gradient of 0.0–1.0 M NaCl. The resulting fractions containing IPAF (Fig. 1B) were then dialyzed in PBS and concentrated to appropriate conditions.

### Electrophoresis analysis and western blotting

Purified IPAF was analyzed by SDS-PAGE with a Bio-Rad mini protein III gel apparatus (Bio-Rad, Hercules, CA). Molecular weight of IPAF was determined on the basis of the migration on gel relatively to pre-stained protein marker (GE Healthcare). The gels were stained with Coomassie brilliant blue R250 (CBR) to visualize protein or with periodic acid-Schiff (PAS) reagent to determine carbohydrate contents. Purified IPAF was separated by SDS-PAGE and transferred onto polyvinylidene difluoride (PVDF) Immobilon P (Millipore, Billerica, MA) membrane using Trans-Blot Cell system (Bio-Rad) in transfer buffer. The PVDF membrane was blocked with PBS containing 5% BSA (Sigma-Aldrich St. Louis, MO) at 4°C overnight. The PVDF membrane was then subjected to western blotting with anti-IPAF monoclonal antibody produced in our laboratory.

### Gel – filtration capillary electrophoresis analysis

Two sets of IPAF samples, reduced and non-reduced, were prepared for gel-filtration capillary electrophoresis (GFCE) analysis using the SDS-MW Analysis Kit (Beckman Coulter, Brea, CA). For the reduced IPAF, protein concentration was adjusted to 0.5 mg/mL with sample buffer, 2 µL of internal standard (synthetic peptide with MW of 10 kDa) and 5 µg of 2-mercaptoethanol was added to 95 µL of protein sample. The sample was then heated in water bath at 100°C for 3 min. The non-reduced IPAF was prepared by adjusting protein concentration to 0.5 mg/mL with sample buffer, and adding 2 µL of internal standard to 95 µL of protein sample. The sample was subsequently heated in water bath at 65°C for 3 min. The protein size standard (synthetic peptides with MW of 10, 20, 35, 50, 100 kDa) was prepared following manufacturer's instructions. The size standard and samples were then loaded onto PA 800 plus Pharmaceutical Analysis System (Beckman Coulter, Brea, CA) for GFCE analysis. The results were analyzed with 32 Karat™ Version 9.0 and PA 800 plus Software. The MW was derived by normalizing the migration time of the samples with the 10 kDa internal standard, and calibrated with the standard curve constructed with the protein size standard.

### N-terminal amino acid sequence analysis

Purified IPAF was separated by SDS-PAGE and transferred onto PVDF membrane using Trans-Blot Cell system (Bio-Rad) in transfer buffer. The protein band corresponding to IPAF was cut from CBR stained membrane and subjected to Edman degradation. Automated Edman degradation and sequence analysis were carried out on an Applied Biosystems Procise Sequencer (Mission Biotech, Taipei, Taiwan).

### Molecular cloning of IPAF gene

To clone the cDNA of IPAF, total RNA of *A. formosanus* was extracted and RACE was carried out as described thoroughly in our previous report [33]. In brief, total RNA was extracted from *A. formosanus* using Qiagen RNeasy Plant Mini Kit (Qiagen, Germantown, MD) according to the manufacturer's instruction and then utilized to synthesize the template cDNA by employing the Thermoscript RT-PCR system (Invitrogen, Carlsbad, CA). 3′ RACE-PCRs were conducted using 3′-SMART RACE cDNA Amplification Kit (Clontech, Mountain View, CA) and a gene-specific primer a36 (5′-GCCAGTAGTTTAGGNACTGG- 3′) degenerated from the IPAF N-terminal amino acid sequence. A 700 bp PCR product (PRa36 fragment) was then cloned into pGEM-T Easy vector (Promega, Madison, WI) which was subsequently transformed into E. coli DH5R, and the colonies were selected for plasmid DNA extraction and sequencing. The 5′ end of IPAF was acquired by utilizing the 5′-SMART RACE cDNA Amplification Kit (Clontech) with a b18 primer (5′- GACGTTCTGTGGCTTATAAAT – 3′), designed based on the sequence of PRa36 fragment. A 600 bp PCR product (PRb18 fragment) was cloned and sequenced via the same method. After aligning PRa36 and PRb18 fragments, the complete sequence of IPAF cDNA was attained. This sequence was amplified and validated by independent PCR cloning using IPAF-specific primer located amid 5′- and 3′-UTR) of IPAF cDNA, and compared for homologous gene using the BLAST program purveyed by NCBI (http://www.ncbi.nlm.nih.gov/BLAST). Multiple alignment of IPAF amino acid sequence with other sequences was conducted by the ClustalW2 [34] program on the website of EMBL-EBI (http://www.ebi.ac.uk/Tools/clustalw2).

### Mice and cell culture

BALB/c mice between 6 to 8 weeks of age were purchased from the National Laboratory Animal Center, Taipei, Taiwan. TLR4 deficient C57BL/10ScN and TLR2 deficient B6.129-TLR2tmlkir/J were purchased from The Jackson Laboratory. The mice were maintained in our animal facility under pathogen-free condition. All animal studies were permitted by the Institutional Animal Care and Use Committee of National Taiwan University (Approval ID: NTU-IACUC-98-112), and performed according to the regulations of the NTU-IACUC. Mice were splenectomized after euthanasia for splenocytes acquisition, cells were suspended in DMEM medium (Hyclone, Logan, Utah) containing 10% heat inactivated fetal bovine serum (FBS) (GIBCO-BRL Life Technologies, New York, NY) and cultured in 96-well microplate (Corning, Lowell, MA) at cell density of 5×10^5^ per well in Hera Cell (Heraeus group, Hanau, Germany) at 37°C with 5% CO_2_ humidified air. T and B lymphocytes were positively selected from total splenocytes by MACS separation system (Miltenyi Biotec, Bergisch Gladbach, Germany) using anti-mouse CD90 and anti-mouse CD19 microbeads, respectively. The purities of T lymphocytes and B lymphocytes were above 95% as analyzed by flow cytometry.

### Fluorescence-activated cell sorting (FACS)

For cell surface markers and MHC class II detection, cells were suspended in FACS buffer containing 0.2% (v/v) FBS and 0.01% (w/w) sodium azide and stained with FITC-labeled anti-mouse CD45R/B220, PE-labeled anti-mouse CD3, PE-labeled anti-mouse CD69, or FITC-labeled anti-mouse MHC class II antibodies (eBioscience, San Diego, CA) at 4°C for 30 minutes in dark. For DNA detection, cells were washed twice and suspended with FACS buffer (150 µL/10^6^ cells) before fixed by 100% ethanol (350 µL/10^6^ cells) at −20°C for 30 minutes and then stained with PI/RNase staining buffer (BD Biosciences, San Jose, CA). Cells were then analyzed with FACScan and these data were acquired by CellQuest software (BD Biosciences). The results were expressed as percentage of positive fluorescent cells.

### Measurement of cell proliferation

The cell proliferation was determined based on the measurement of BrdU uptake by the cells using Cell proliferation ELISA Brd-U kit (Roche, Basel, Switzerland) following manufacturer's instructions. The results were acquired by microplate reader 3550-UV (Bio-Rad).

### Measurement of immunoglobulin production

The levels of mouse IgM and IgG production in cell culture supernatant were analyzed using Mouse IgM and Mouse IgG ELISA quantification kits (Bethyl Laboratories, Inc, Montgomery, TX.), respectively, according to manufacturer's instructions. The concentrations of IgM and IgG were calculated by comparing with recombinant mouse IgM and IgG as standards. The results were acquired by microplate reader 3550-UV (Bio-Rad).

### Production of anti-IPAF monoclonal antibody

BALB/c mice were immunized by monthly intraperitoneal injections of 50 µg purified IPAF, which was mixed with an appropriate volume of aluminum potassium sulfate (10% w/v). Splenocytes from immunized mice were fused 3 days after an intraperitoneal injection of immunogen with mouse X63Ag8.653 myeloma cells from the American Type Culture Collection (ATCC; Manassas, VA) using 50% polyethylene glycol 1500 (Roche). Hybridoma was selected in a hypoxanthine-aminopterin-thymidine medium (Gibco/BRL Life Technologies, Eggenstein, Germany) and the supernatants were screened by conventional immunoenzymatic assays using purified IPAF as a tracer. The cells from the positive wells were cloned twice using limiting dilution method. Monoclonal antibodies were purified from the supernatant of hybridoma culture by protein A/G affinity chromatography (GE Healthcare).

### Data analysis

All experimental results are presented as mean ± SD of three independent experiments performed in triplicates (n = 3). Statistical comparisons were performed by one way ANOVA using PC-SAS (Statistical Analysis System, SAS Institute., USA) and differences between experimental data were determined to be significant at *P* value<0.05.

## Supporting Information

Supporting Information S1 Induction of TNF-α production in RAW 264.7 macrophages by active protein fraction eluted with DEAE-52 column. RAW 264.7 macrophages were cultured in 96-well plate and stimulated with 0, 10, 20, 30, and 40 µg/ml active protein fraction eluted with DEAE-52 column for 24 hours. TNF-α concentration in the culture supernatant was determined by ELISA using mouse recombinant TNF-α as standard.
(TIF)

Supporting Information S2 SDS-PAGE analysis of the active protein fractions eluted with DEAE-52 column. The active protein fractions eluted with DEAE-52 column were analyzed by SDS-PAGE 20 µl of fraction 8, 10, 12, 14, 16, 18, 20, 22, and 24 were loaded in lane 2, 3, 4, 5, 6, 7, 8, 9, and 10 respectively, and molecular weight of protein was determined by comparison between prestained protein marker.
(TIF)

Supporting Information S3 IPAF stimulated TNF-α secretion in RAW264.7 macrophages. RAW 264.7 cells were cultured in 96-well plate (5×10^5^ cells/well) and treated with indicated concentrations of IPAF for 24 hours. TNF-α concentration in the culture supernatant was determined by ELISA using mouse recombinant TNF-α as standard.
(TIF)

Supporting Information S4 IPAF Stimulated IFN-γ secretion in mouse splenocytes. Splenocytes harvested from BALB/c were treated with or without 8 µg/ml IPAF for 72 hours. IFN-γ concentration in the culture supernatant was determined by ELISA using mouse recombinant IFN-γ as standard.
(TIF)

Supporting Information S5 Alignment of the 3′-RACE and 5′-RACE products and the deduced amino acid sequences. 3′-RACE PCR was performed using a gene specific primer a36 (red arrow), which was degenerated from the N-terminal amino acid sequence (green). By taking advantage of the PRa36 sequence, primer b18 (blue arrow) was designed and by which the 5′-RACE PCR was conducted. The complete nucleotide sequence of IPAF cDNA was obtained through merging the corresponding sequences of PRa36 and PRb18.
(TIF)

Supporting Information S6 Signal peptide prediction of IPAF. Putative signal peptide was predicted by SignalP prediction program Using neural networks (NN) and hidden Markov models (HMM) trained on eukaryotes.
(TIF)

Supporting Information S7 Hemagglutination assay of IPAF on human and mouse erythrocyte. IPAF and fungal immunomodulatory protein (FIP)-fve were adjusted to 1 mg/mL with PBS, and two-fold serial dilution was performed. 100 µL/well of protein sample was placed in U bottom 96-well plate, and 50 µL of 3% erythrocyte solution was added to each well. The plate was incubated at room temperature for 1 hour and the result was recorded with a digital camera. FIP-fve served as positive control where the positive wells were clouded due to hemagglutination. The erythrocyte in the IPAF solution all precipitated within an hour, leaving a clear zone around the erythrocyte pellet.
(TIF)

Supporting Information S8 The involvement of TLR4 in the IPAF-induced activation of murine peritoneal macrophages. Peritoneal macrophages isolated from C57BL/6 and C57BL/10ScN (TLR4^−/−^) mice were treated with 8 µg/ml IPAF for the indicated period, and TNF-α concentrations were determined by ELISA using recombinant mouse TNF-α as standard.
(JPG)

Supporting Information S9 IPAF enhanced CD69 expression in splenic B lymphocytes of C57BL/10ScN. B lymphocytes purified from C57BL/10ScN, incubated in 96-well plate (3×10^5^ cells per well), and stimulated with 30 µg/ml IPAF, 10 µg/ml LPS or medium alone for 24 hours. Cells were then stained with PE-labeled anti-mouse CD69 for flow cytometry analysis. Data are presented as percentage of CD69^+^ cells in total cells.
(TIF)

Supporting Information S10 IPAF enhanced MHC class II expression in splenic B lymphocytes of C57BL/10ScN. B lymphocytes purified from C57BL/10ScN, incubated in 96-well plate (3×10^5^ cells per well), and stimulated with 30 µg/ml IPAF, 10 µg/ml LPS or 200 ng/ml IFN-γ or medium alone for 24 hours. Cells were then stained with FITC-labeled anti-mouse MHC class II for flow cytometry analysis. Data are presented as percentage of MHC II^+^ cells in total cells.
(TIF)

Supporting Information S11 IPAF enhanced CD69 expression in splenic B lymphocytes of B6.129-TLR2tmlkir/J. B lymphocytes purified from B6.129-TLR2tmlkir/J, incubated in 96-well plate (3×10^5^ cells per well), and stimulated with 30 µg/ml IPAF, 10 µg/ml LPS or 200 ng/ml IFN-γ or medium alone for 24 hours. Cells were then stained with PE-labeled anti-mouse CD69 for flow cytometry analysis. Data are presented as percentage of CD69^+^ cells in total cells.
(TIF)

Supporting Information S12 IPAF enhanced MHC class II expression in splenic B lymphocytes of B6.129-TLR2tmlkir/J. B lymphocytes purified from B6.129-TLR2tmlkir/J, incubated in 96-well plate (3×10^5^ cells per well), and stimulated with 30 µg/ml IPAF, 10 µg/ml LPS or 200 ng/ml IFN-γ or medium alone for 24 hours. Cells were then stained with FITC-labeled anti-mouse MHC class II for flow cytometry analysis. Data are presented as percentage of MHC II^+^ cells in total cells.
(TIF)
