# Supplementary figures and images for: Molecular Cloning of a New Immunomodulatory Protein from Anoectochilus formosanus which Induces B Cell IgM Secretion through a T-Independent Mechanism
Source: PLoS One. 2011 Jun 16;6(6):e21004. doi: 10.1371/journal.pone.0021004 (PMC3116865; doi:10.1371/journal.pone.0021004)

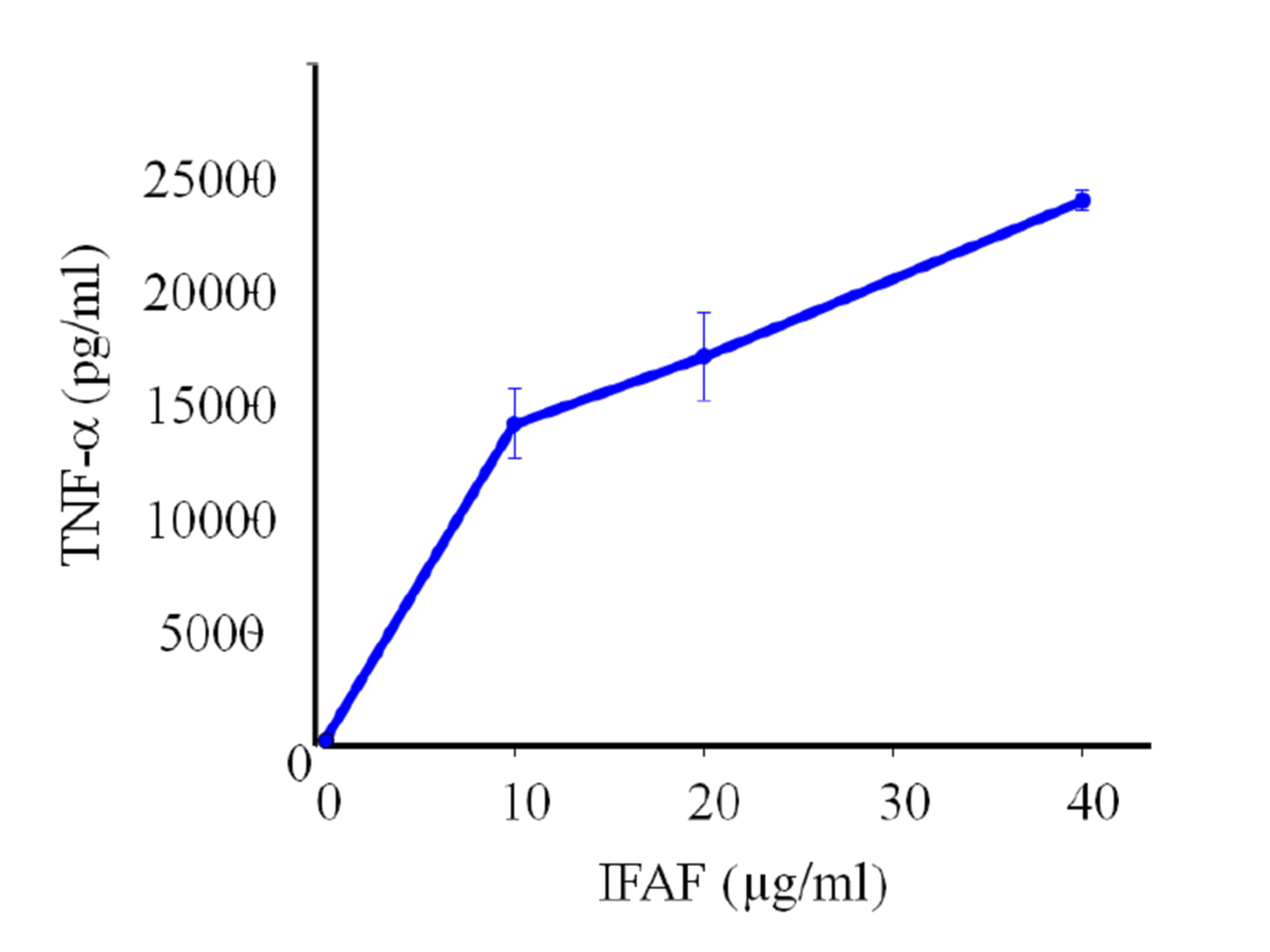

Supplement: Supporting Information S1 — Induction of TNF-α production in RAW 264.7 macrophages by active protein fraction eluted with DEAE-52 column. RAW 264.7 macrophages were cultured in 96-well plate and stimulated with 0, 10, 20, 30, and 40 µg/ml active protein fraction eluted with DEAE-52 column for 24 hours. TNF-α concentration in the culture supernatant was determined by ELISA using mouse recombinant TNF-α as standard. (TIF) [file pone.0021004.s001.tif]

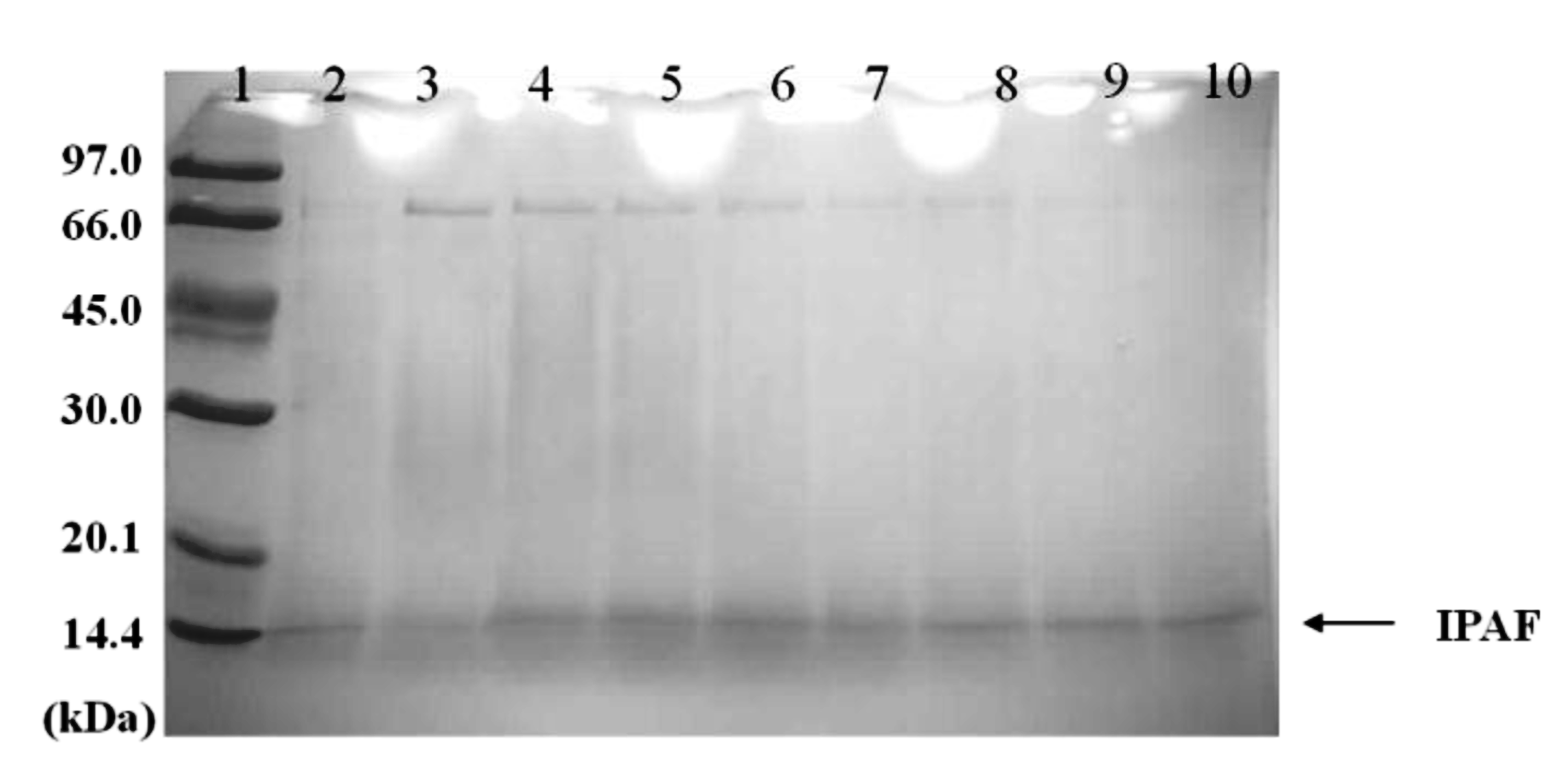

Supplement: Supporting Information S2 — SDS-PAGE analysis of the active protein fractions eluted with DEAE-52 column. The active protein fractions eluted with DEAE-52 column were analyzed by SDS-PAGE 20 µl of fraction 8, 10, 12, 14, 16, 18, 20, 22, and 24 were loaded in lane 2, 3, 4, 5, 6, 7, 8, 9, and 10 respectively, and molecular weight of protein was determined by comparison between prestained protein marker. (TIF) [file pone.0021004.s002.tif]

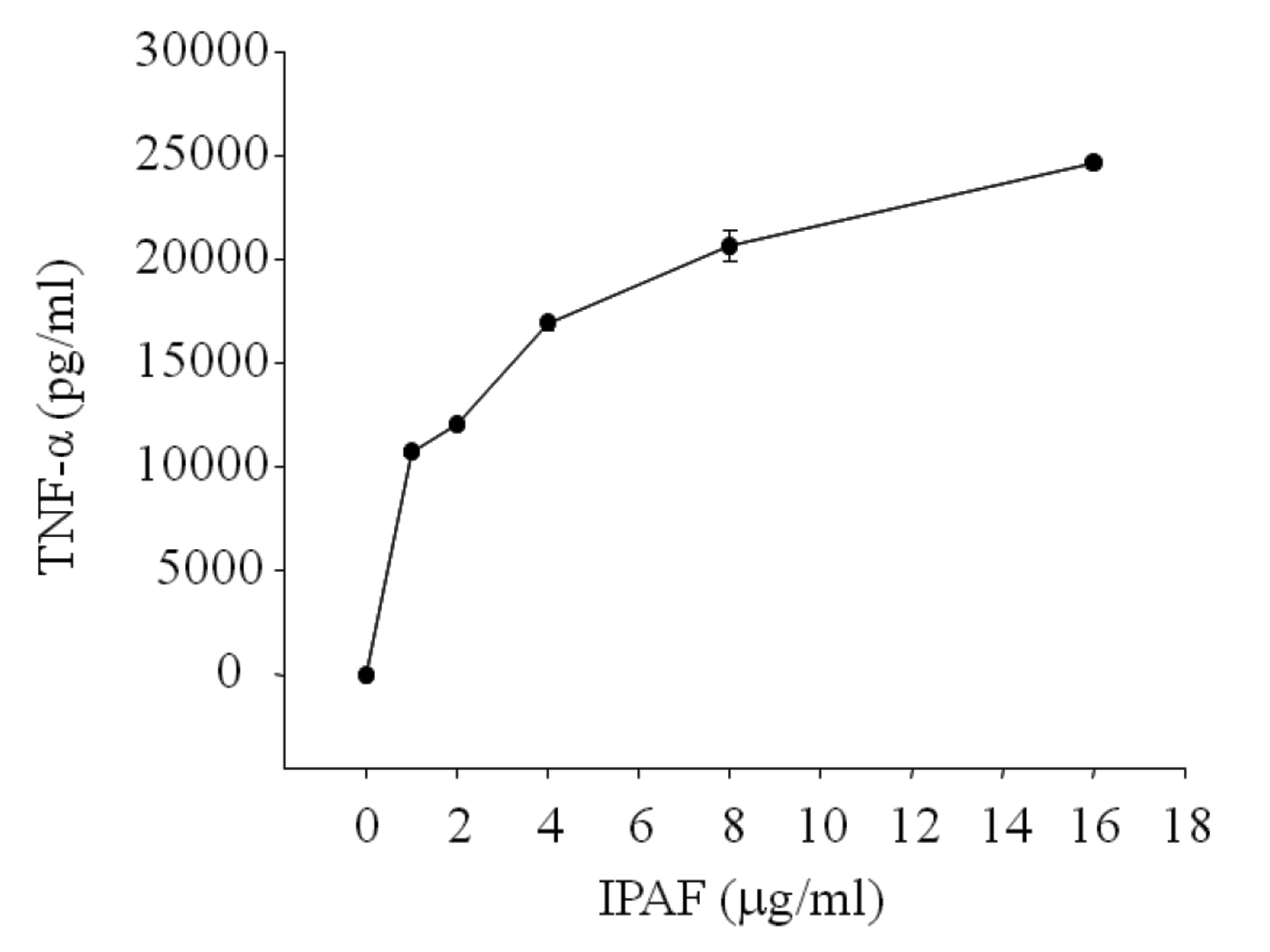

Supplement: Supporting Information S3 — IPAF stimulated TNF-α secretion in RAW264.7 macrophages. RAW 264.7 cells were cultured in 96-well plate (5×10^5^ cells/well) and treated with indicated concentrations of IPAF for 24 hours. TNF-α concentration in the culture supernatant was determined by ELISA using mouse recombinant TNF-α as standard. (TIF) [file pone.0021004.s003.tif]

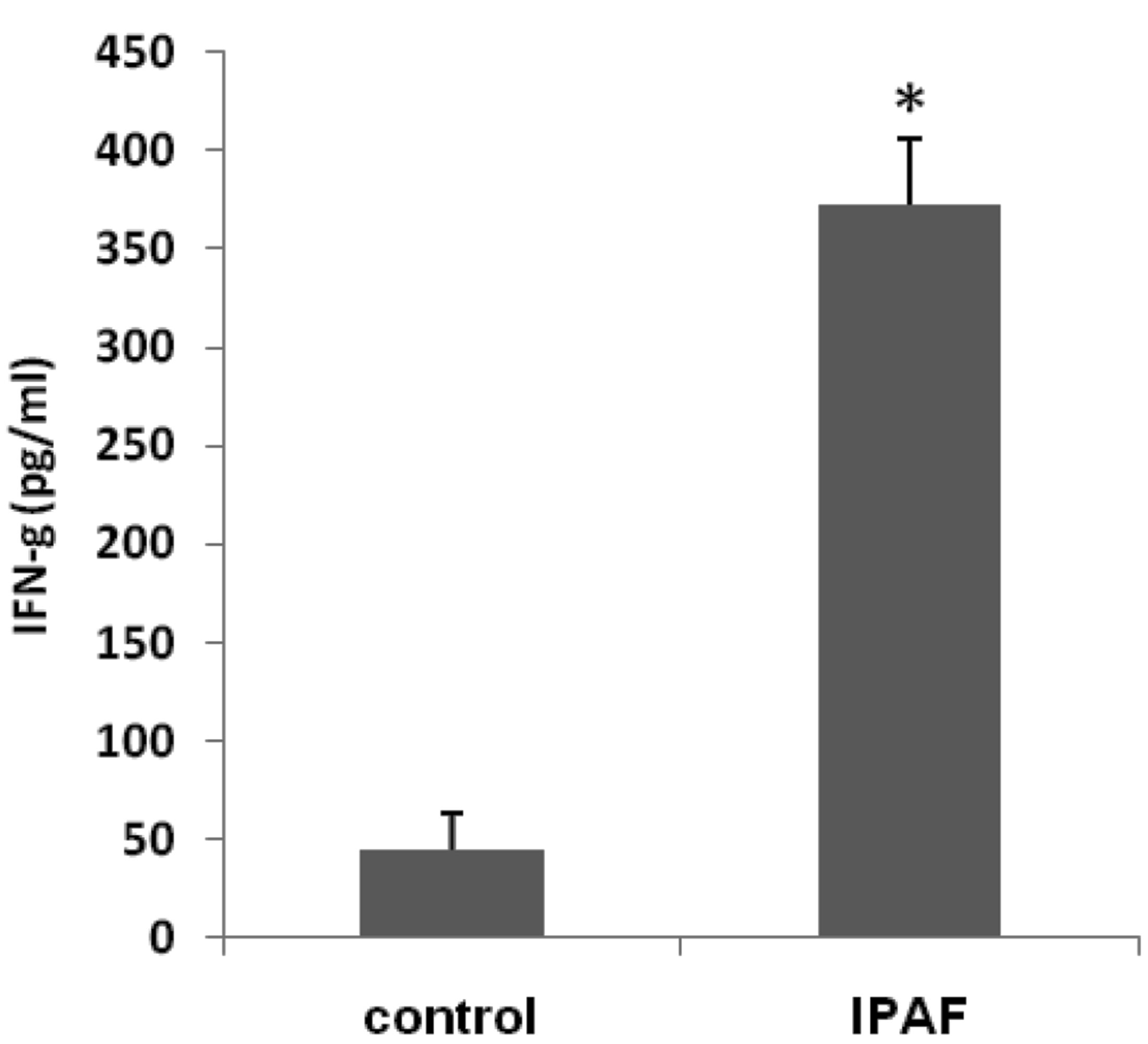

Supplement: Supporting Information S4 — IPAF Stimulated IFN-γ secretion in mouse splenocytes. Splenocytes harvested from BALB/c were treated with or without 8 µg/ml IPAF for 72 hours. IFN-γ concentration in the culture supernatant was determined by ELISA using mouse recombinant IFN-γ as standard. (TIF) [file pone.0021004.s004.tif]

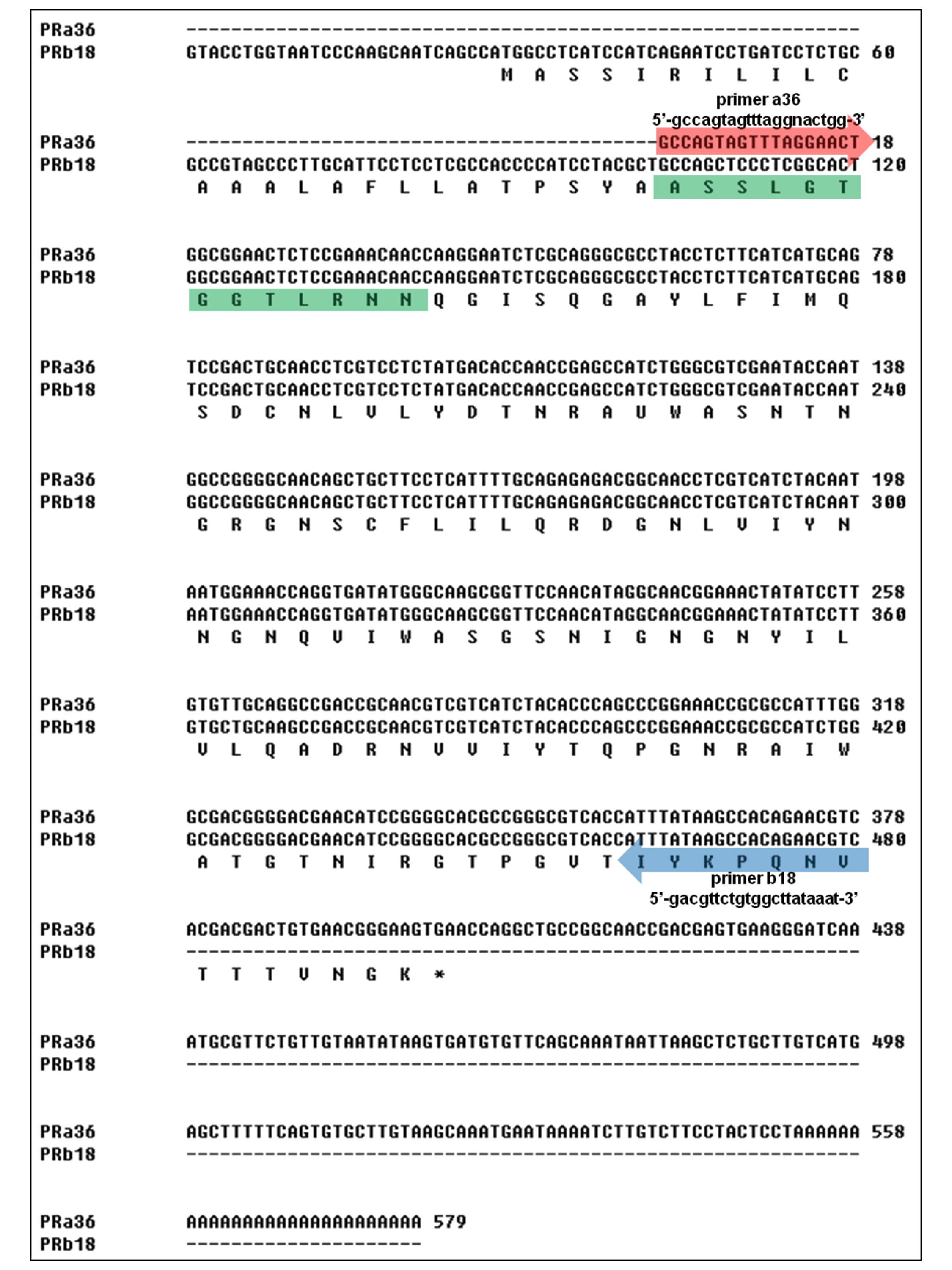

Supplement: Supporting Information S5 — Alignment of the 3′-RACE and 5′-RACE products and the deduced amino acid sequences. 3′-RACE PCR was performed using a gene specific primer a36 (red arrow), which was degenerated from the N-terminal amino acid sequence (green). By taking advantage of the PRa36 sequence, primer b18 (blue arrow) was designed and by which the 5′-RACE PCR was conducted. The complete nucleotide sequence of IPAF cDNA was obtained through merging the corresponding sequences of PRa36 and PRb18. (TIF) [file pone.0021004.s005.tif]

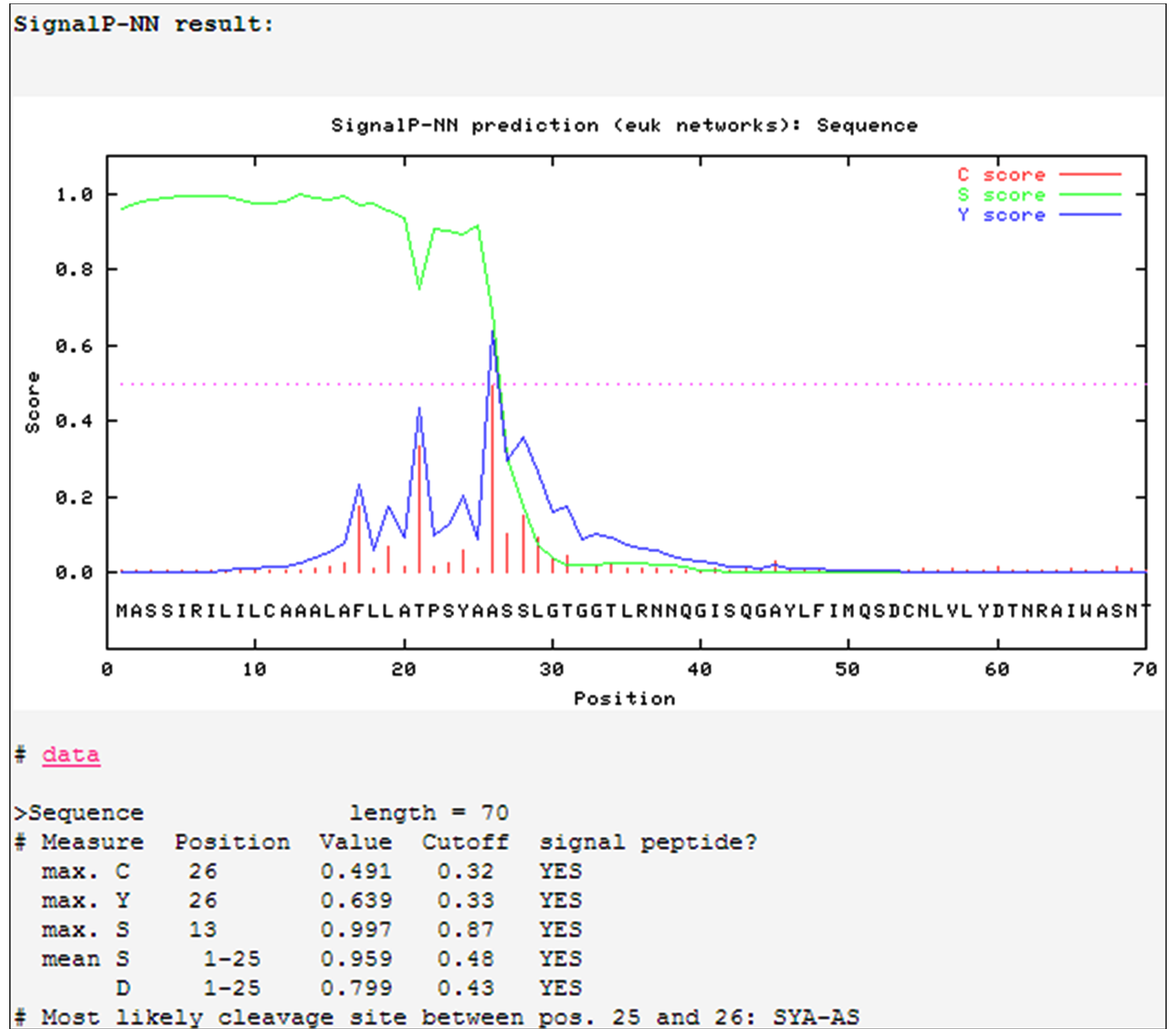

Supplement: Supporting Information S6 — Signal peptide prediction of IPAF. Putative signal peptide was predicted by SignalP prediction program Using neural networks (NN) and hidden Markov models (HMM) trained on eukaryotes. (TIF) [file pone.0021004.s006.tif]

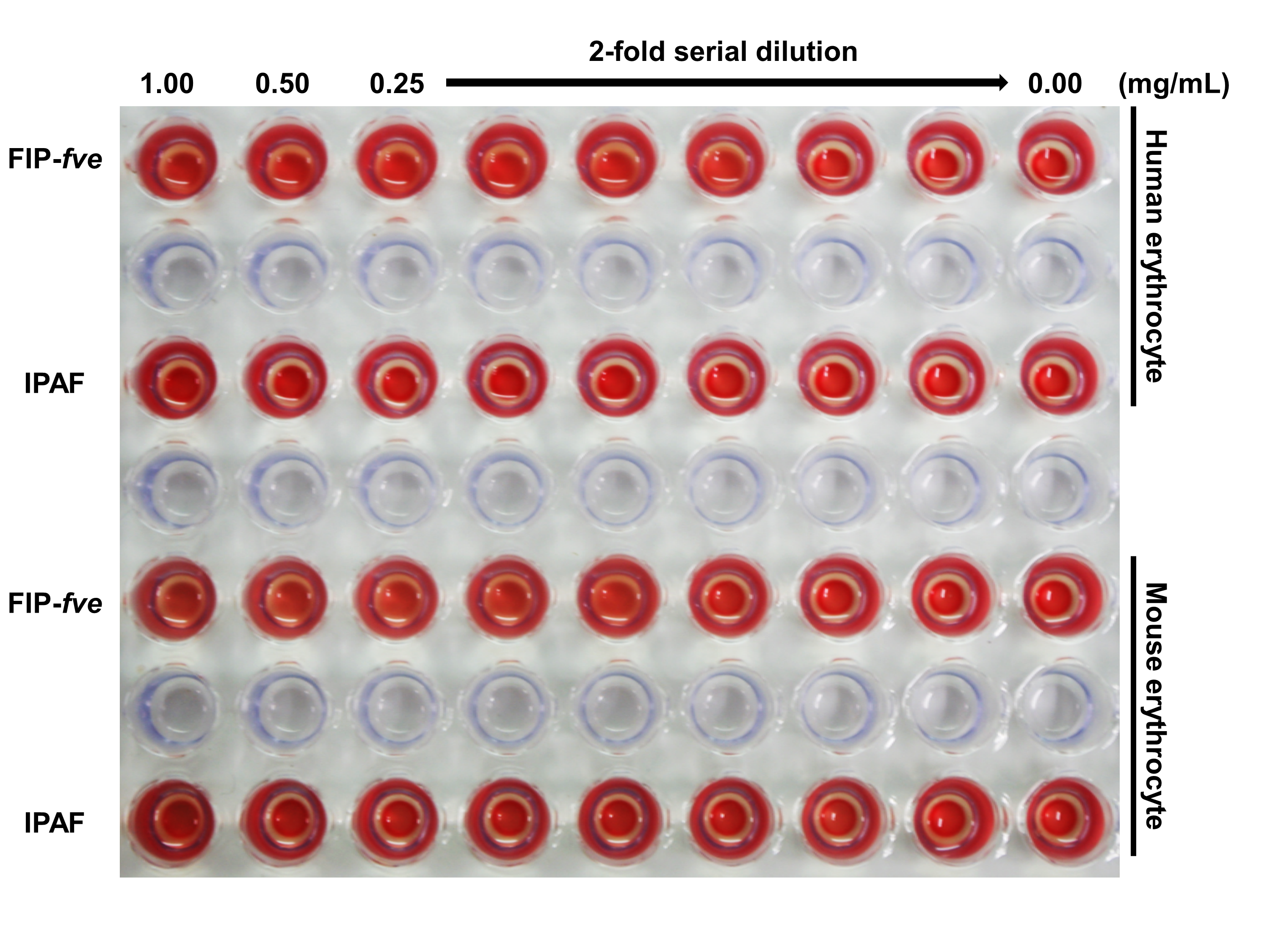

Supplement: Supporting Information S7 — Hemagglutination assay of IPAF on human and mouse erythrocyte. IPAF and fungal immunomodulatory protein (FIP)-fve were adjusted to 1 mg/mL with PBS, and two-fold serial dilution was performed. 100 µL/well of protein sample was placed in U bottom 96-well plate, and 50 µL of 3% erythrocyte solution was added to each well. The plate was incubated at room temperature for 1 hour and the result was recorded with a digital camera. FIP-fve served as positive control where the positive wells were clouded due to hemagglutination. The erythrocyte in the IPAF solution all precipitated within an hour, leaving a clear zone around the erythrocyte pellet. (TIF) [file pone.0021004.s007.tif]

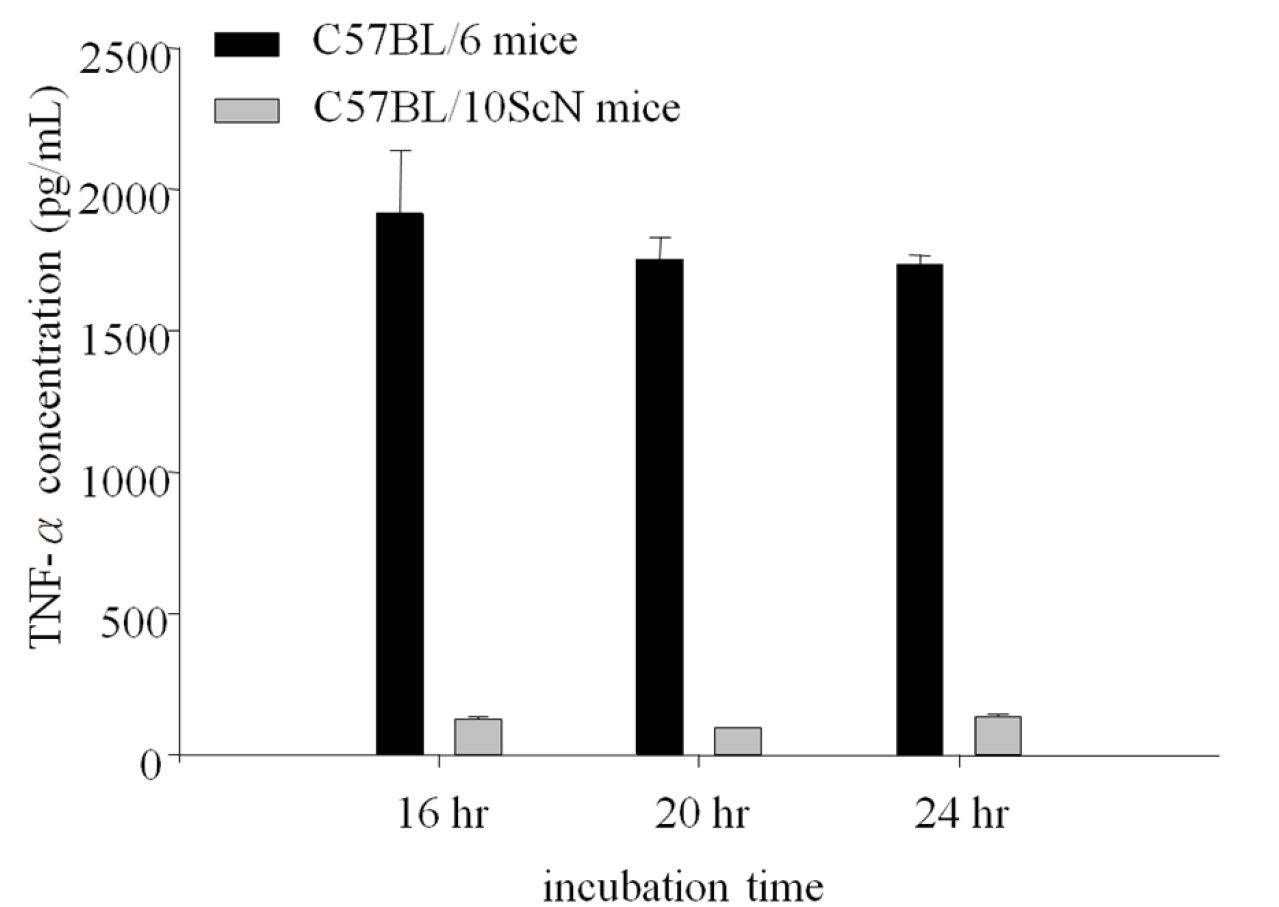

Supplement: Supporting Information S8 — The involvement of TLR4 in the IPAF-induced activation of murine peritoneal macrophages. Peritoneal macrophages isolated from C57BL/6 and C57BL/10ScN (TLR4−/−) mice were treated with 8 µg/ml IPAF for the indicated period, and TNF-α concentrations were determined by ELISA using recombinant mouse TNF-α as standard. (JPG) [file pone.0021004.s008.jpg]

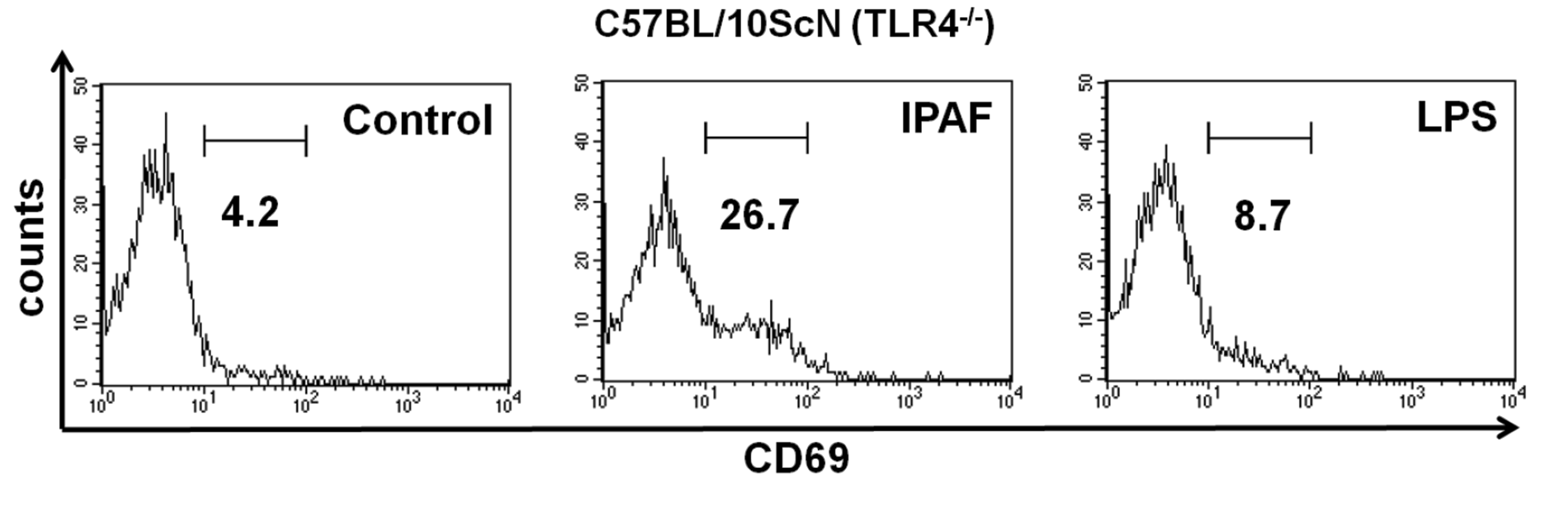

Supplement: Supporting Information S9 — IPAF enhanced CD69 expression in splenic B lymphocytes of C57BL/10ScN. B lymphocytes purified from C57BL/10ScN, incubated in 96-well plate (3×10^5^ cells per well), and stimulated with 30 µg/ml IPAF, 10 µg/ml LPS or medium alone for 24 hours. Cells were then stained with PE-labeled anti-mouse CD69 for flow cytometry analysis. Data are presented as percentage of CD69+ cells in total cells. (TIF) [file pone.0021004.s009.tif]

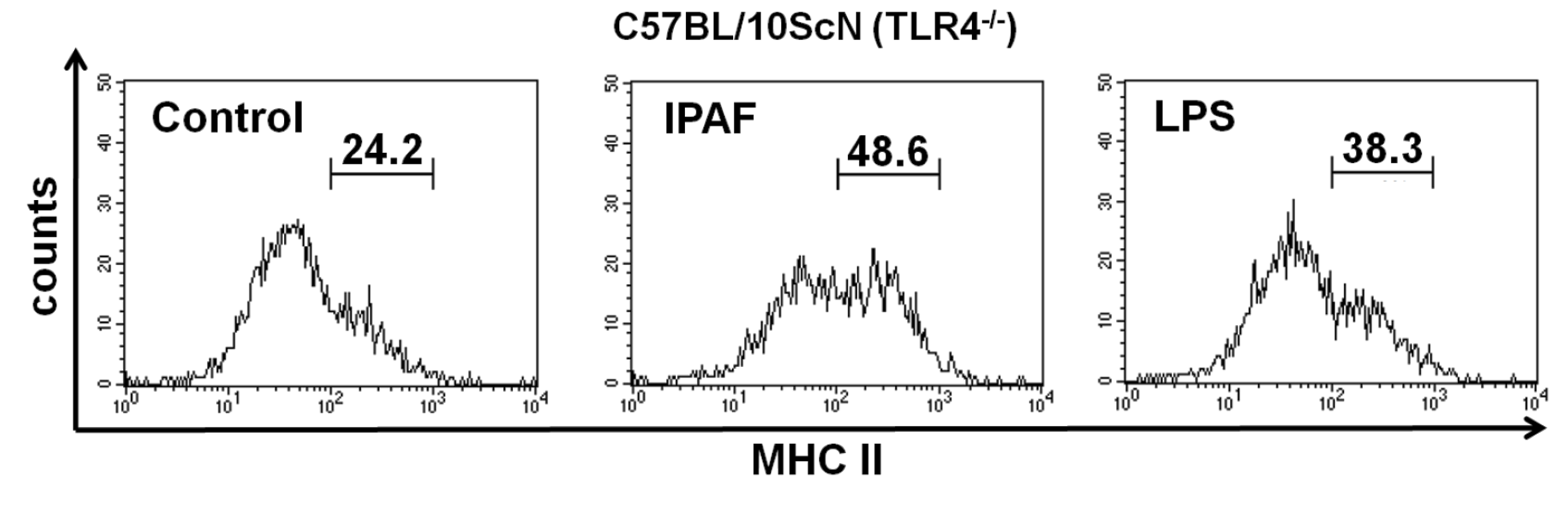

Supplement: Supporting Information S10 — IPAF enhanced MHC class II expression in splenic B lymphocytes of C57BL/10ScN. B lymphocytes purified from C57BL/10ScN, incubated in 96-well plate (3×10^5^ cells per well), and stimulated with 30 µg/ml IPAF, 10 µg/ml LPS or 200 ng/ml IFN-γ or medium alone for 24 hours. Cells were then stained with FITC-labeled anti-mouse MHC class II for flow cytometry analysis. Data are presented as percentage of MHC II+ cells in total cells. (TIF) [file pone.0021004.s010.tif]

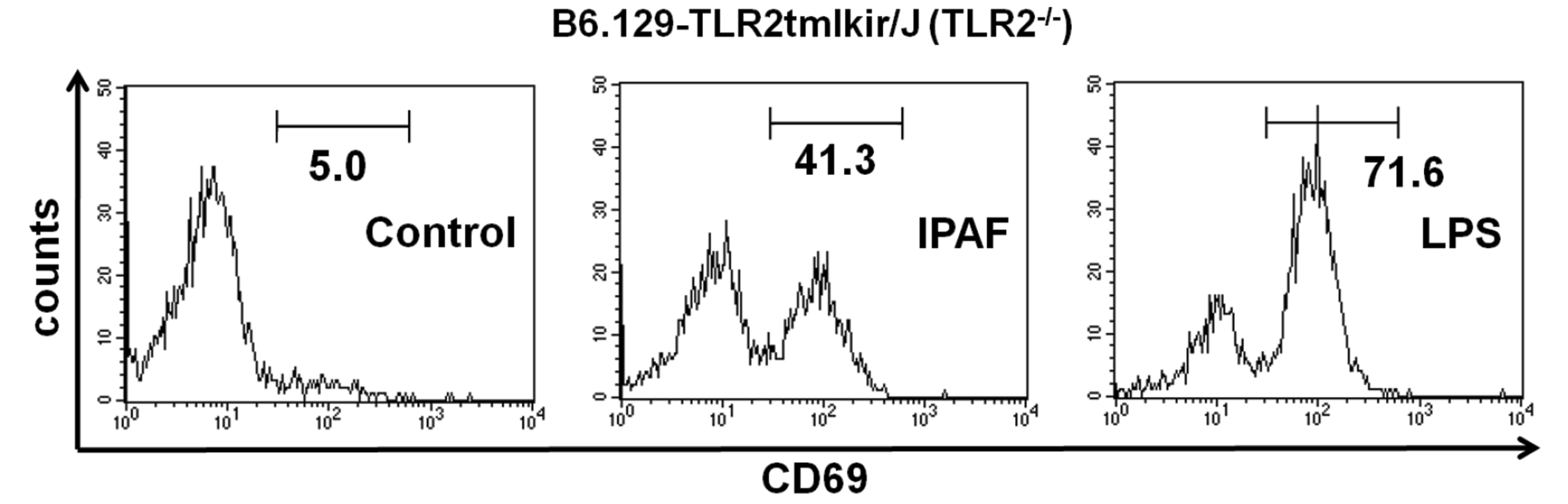

Supplement: Supporting Information S11 — IPAF enhanced CD69 expression in splenic B lymphocytes of B6.129-TLR2tmlkir/J. B lymphocytes purified from B6.129-TLR2tmlkir/J, incubated in 96-well plate (3×10^5^ cells per well), and stimulated with 30 µg/ml IPAF, 10 µg/ml LPS or 200 ng/ml IFN-γ or medium alone for 24 hours. Cells were then stained with PE-labeled anti-mouse CD69 for flow cytometry analysis. Data are presented as percentage of CD69+ cells in total cells. (TIF) [file pone.0021004.s011.tif]

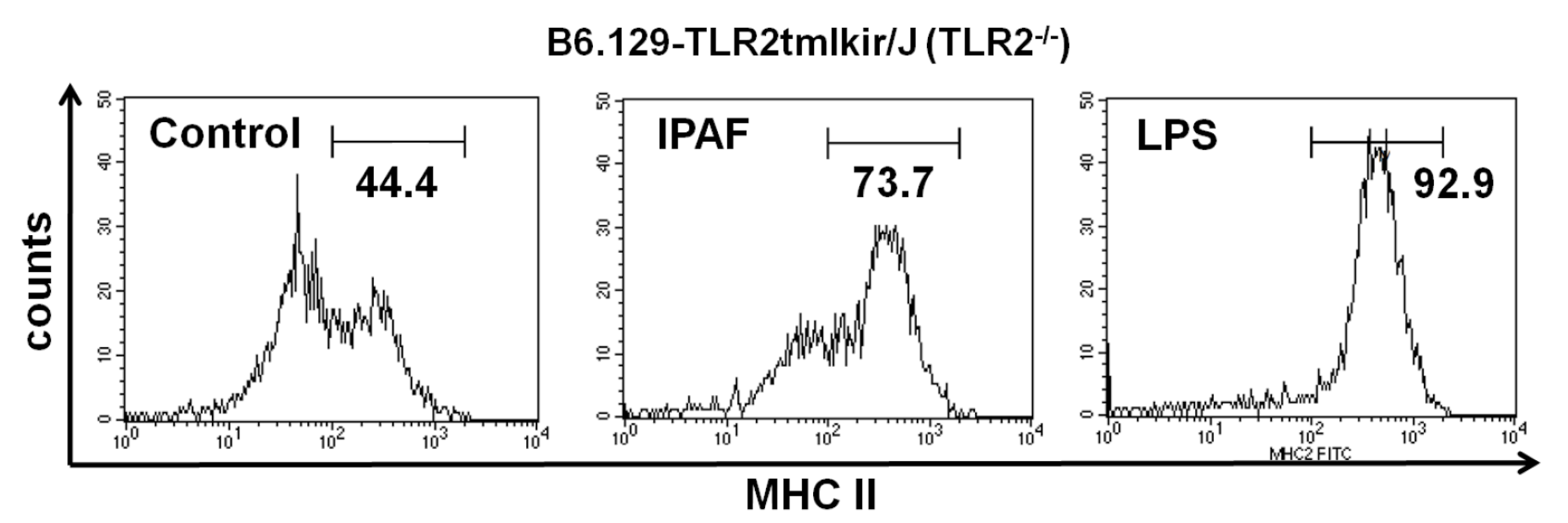

Supplement: Supporting Information S12 — IPAF enhanced MHC class II expression in splenic B lymphocytes of B6.129-TLR2tmlkir/J. B lymphocytes purified from B6.129-TLR2tmlkir/J, incubated in 96-well plate (3×10^5^ cells per well), and stimulated with 30 µg/ml IPAF, 10 µg/ml LPS or 200 ng/ml IFN-γ or medium alone for 24 hours. Cells were then stained with FITC-labeled anti-mouse MHC class II for flow cytometry analysis. Data are presented as percentage of MHC II+ cells in total cells. (TIF) [file pone.0021004.s012.tif]
